# Electrophysiological correlates of unconscious processes of race

**DOI:** 10.1038/s41598-021-91133-2

**Published:** 2021-06-02

**Authors:** Francesca Pesciarelli, Irene Leo, Luana Serafini

**Affiliations:** 1 Department of Biomedical, Metabolic, and Neural Sciences, University of Modena and Reggio Emilia, Via Campi 287, 41100 Modena, Italy; 2 Department of Developmental Psychology and Socialization, University of Padova, Padova, Italy

**Keywords:** Human behaviour, Cognitive neuroscience, Consciousness

## Abstract

The study aimed to examine the neural mechanisms underlying implicit other-race face processing by the use of the masked and unmasked priming manipulation. Two types of prime-target pairs were presented while recording Event-related potentials (ERPs): Same face pairs (prime-target were identical faces), and Different face pairs (prime-target were different faces). Prime-target pairs were half Asian (other-race) and half Caucasian (own-race) faces. The face stimuli on each pair were of the same gender and race. Participants (all Caucasians) had to decide whether the target was a male or a female face (gender task). The prime face could be unmasked or masked. On the behavioral side, our findings showed a race effect, that is slower reaction times (RTs) for other-race than own-race face stimuli, regardless of masking. On the ERPs side, our data showed a race effect across all components analyzed (P100, N100, N200, P300), under both the unmasked and masked manipulations. Besides, we found, in the unmasked condition, a priming effect as a function of race on the N100, N200, and P300 components; but, interestingly, in the masked condition, only on the P300. Overall, our findings provide evidence that race information is available very early in the brain and can strongly activate and influence people’s behaviors even without conscious awareness.

## Introduction

The race effect (Other-Race Effect, ORE) is a set of occurrences in which the faces of one's own ethnic group are processed differently from those belonging to other ethnic groups. In general, adults recognize and process faces belonging to their race faster and more accurately, while error rates increase considerably and the stimulus processing is slower when they have to recognize faces belonging to a different ethnic group from their own^1^. The ORE has been shown in many studies on face recognition between different ethnic groups (e.g.^2^) and different age groups (e.g.^3^). Two different accounts have been proposed to explain the ORE: The Experienced based holistic account, EBH^4^, and the Sociocognitive account^5^. The first argues that the perception of the face is a high-level visual process, resulting from the interaction between incoming information and internal representations and that to explain it, it is necessary to understand the nature and characteristics of these interactions. Sociocognitive theory, on the other hand, argues that the ORE is a phenomenon that is totally independent of how people process and represent a face and that it is rather due to social, motivational, and attentive aspects. The current research aimed to shed light on this theoretical debate by investigating the brain mechanisms and the temporal course of the implicit/automatic own- and other-race face processing. For this purpose, the masked priming manipulation has been utilized, a manipulation that allows investigating the unconscious processing of a stimulus^6–9^. In this paradigm, the prime stimuli are displayed very briefly and are then obscured/masked either by a series of letters or symbols or directly by the target stimuli. Participants generally report not having seen the prime stimulus and respond to the target faster and more accurately when prime-target are identical or semantically related (e.g., dog–dog/cat–dog) compared to when they are not (e.g., shoe–dog). Although the mechanisms underlying these unconscious effects are under debate^10,11^, consensus exists that the masked priming paradigm reflects implicit/automatic mechanisms of stimulus processing^9^.

We, therefore, preferred this paradigm to other experimental designs since it is a suitable tool for exploring the brain mechanisms involved in the unconscious processing of the face and the stages involved in the activation of race information^12–16^. Specifically, if any differences were to be found between own-race and other-race face priming effects (same vs. different prime-target pairs), these effects could not be explained by strategic processing. This could rule out a stream of the sociocognitive account of the ORE arguing for a strategically/intentionally deeper processing of own-race faces (e.g.^5,17,18^). For instance, it is hypothesized that due to racial attitudes or low interest, observers would not be motivated to process or to pay attention to other-race faces. On the contrary, these effects could only be explained as automatic and implicit effects. Since the prime is thought to pre-activate representations in long-term memory which facilitate their later access by the target, differences in priming effects between own-race and other-race faces could reflect less precise and/or less accessible memory representations of other-race than own-race faces, in line with an EBH account. In this vein, different priming effects for own-race and other-race faces could fit with the EBH account. However, some theories within the sociocognitive framework could also be plausible. For instance, social factors such as social categorization (i.e., the classification of social stimuli as ingroup and outgroup) and attentional factors, such as attention capture could also occur automatically (e.g.^19,20^), and thus could also explain different priming effects as a function of race.

To our knowledge, the neural mechanisms underlying race processing have never been explored comparing face stimuli presented above and below threshold. Event-related potentials (ERPs) are particularly suitable because they provide a continuous measurement between the target stimulus and the response, allowing to isolate the effect of a single experimental manipulation at a specific processing stage. Previous ERP studies have concentrated on various ERP waveforms that seem to distinguish own- from other-race face processing (e.g.^21–30^). The differences emerged around 100 ms after presentation of the face stimulus: a positive activity (P100) and a following negative activity (N100), with larger amplitude P100s (^23^; but see^31^) and N100s (^25,32^; but see^26^) evoked for other-race faces. Considering that, the P100 and N100 components are thought to reflect low-level feature processing, their sensitivity to race seems to indicate that information related to race is available early in face processing before perceptual processes are complete. However, the role of these two components on race processing is still under debate, and the contradicting results make it difficult to formulate clear hypotheses.

Another ERP component, the N200, a negative activity peaking between 180 and 300, has been consistently associated with race processing. The N200 is generally modulated by attentional deployment. Research on face processing has shown its sensitivity to faces’ race, expressed in more negative waveforms for faces from own-race than other-race^25,26,32,33^. This greater attention to faces of the own-race has been suggested to reflect automatic processing of deeper levels of attention towards ingroup members. This N200 own-race effect follows initial greater attention to outgroup members showed by the P100 and N100 effects.

Besides, several studies showed a P300 amplitude and latency modulation related to race processing, such that the P300 is larger for other-race compared to own-race faces^25,34–37^. This P300 effect has been taken as reflecting more attention allocated to faces of other-race in comparison of own-race groups^38^. The P300 amplitude has been consistently associated with the task complexity^39^, the stimulus relevance^40^, and the amount of attention directed to the stimulus, while the P3 latency has been associated with the duration of the evaluation of the stimulus when is required a two-choice RTs (e.g.^41^). This P300 other-race effect has been related to contextual updates along relevant features.

To evaluate whether race processing is unconsciously activated, we used the masked priming paradigm. The primes were faces that differed in their race, Caucasian (own-race) or Asian (other-race). The prime stimulus was presented very briefly (33 ms). In the masked manipulation the prime face was preceded and followed by a scrambled face; while in the unmasked manipulation, these scrambled faces were replaced by a black screen, making the prime stimulus fully visible. The prime stimulus was then followed by a Caucasian (own-race) or Asian (other-race) target face. The face stimuli on each pair were of the same gender and race. RTs and EEG were recorded. If our hypothesis that race is processed outside of people’s awareness is correct, we expect race to be processed also in the masked manipulation. Moreover, considering that participants are instructed to attend to gender and not explicitly to race, we can also evaluate responses to index the degree to which the information related to race is implicitly processed.

The present research investigated the time course of the implicit processing of the face, analyzing the early (P100 and N100) and late (N200 and P300) ERP components, in particular examining how the roles of race and priming (intended as a same/different situation), can interact in different stages of face processing. In specific, race (own vs. other) was expected to interact with priming (same: identical faces vs. different: non-identical faces [but of the same gender and race]) under masked and unmasked conditions in a gender-classification task if face identity is processed to a larger degree in faces of the own-race than in faces of the other-race. In this case, we expected a selective (or stronger) identity/priming effect in the own-race condition but not (or weaker) in the other-race condition. If this interaction between race and identity/priming is independent of the participant’s awareness of the faces, the interaction should be found regardless of masking. Besides, this interaction should be found in early ERP components if it relies on early (sensory) processing stages. Few researchers have explored the temporal course of the race effect^22,26,31,42^ and, to the best of our knowledge, none by comparing stimuli proposed above and below threshold. Based on the literature, we hypothesized a masked and unmasked “other race” effect in all the components analyzed and a masked and unmasked priming effect in the late ones. Nevertheless, considering the not consistent data in the literature and the novel approach taken in the present study, it was possible that, for some of the considered ERP components, the priming and race effects could appear in additional time windows.

## Results

### Behavioral results

**Figure 1:**
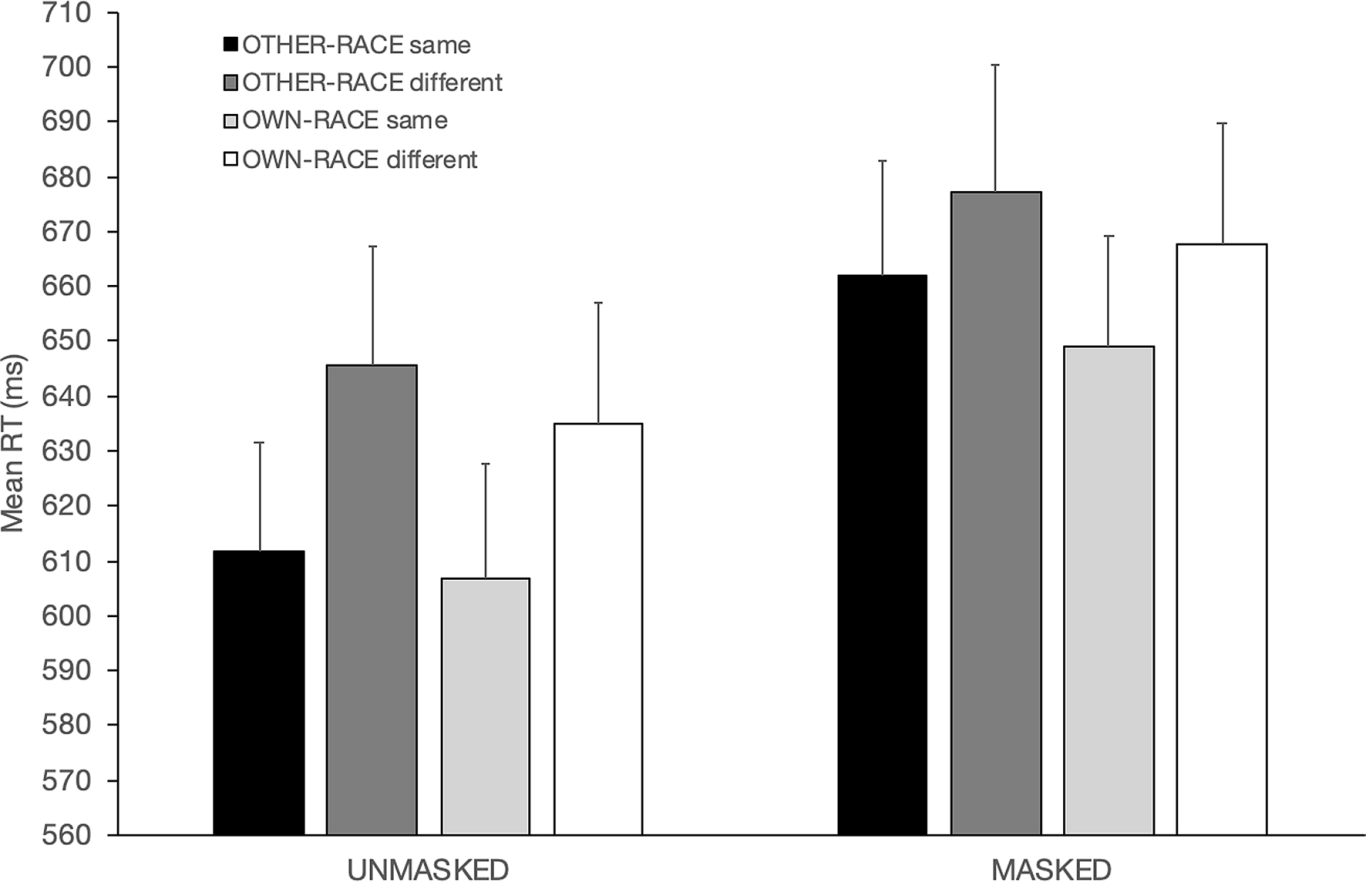
Mean reaction times to target faces separately for the unmasked (left panel) and masked (right panel) procedures as a function of prime-target identity (same vs different) and race (other-race vs own-race). Error bars represent standard errors of the mean.

Figure 1 shows the mean RTs to face targets preceded by same and different face primes. Participants were not able to identify the primes. The omnibus ANOVA performed on the RTs showed a significant main effect of Masking [F(1, 34) = 28.06, p < 0.001, η_p_^2^ = 0.45] revealing faster RTs for the unmasked than masked manipulation; a significant main effect of Race [F(1, 34) = 8.12, p < 0.01, η_p_^2^ = 0.19] revealing faster RTs for own-race faces than other-race faces; a significant main effect of Identity [F(1, 34) = 80.86, p < 0.001, η_p_^2^ = 0.70] revealing faster RTs for the Same than Different condition; and a Masking x Identity interaction [F(1, 34) = 13.98, p < 0.0001, η_p_^2^ = 0.29], revealing a larger priming effect in the unmasked than masked condition, regardless of race. No other effects reached significance (ps > 0.1). The omnibus ANOVA conducted on the accuracy did not reveal any significant effects (ps > 0.05), probably because performance was near ceiling, with all conditions averaging between 94 and 96% correct.

### ERP results

**Figure 2:**
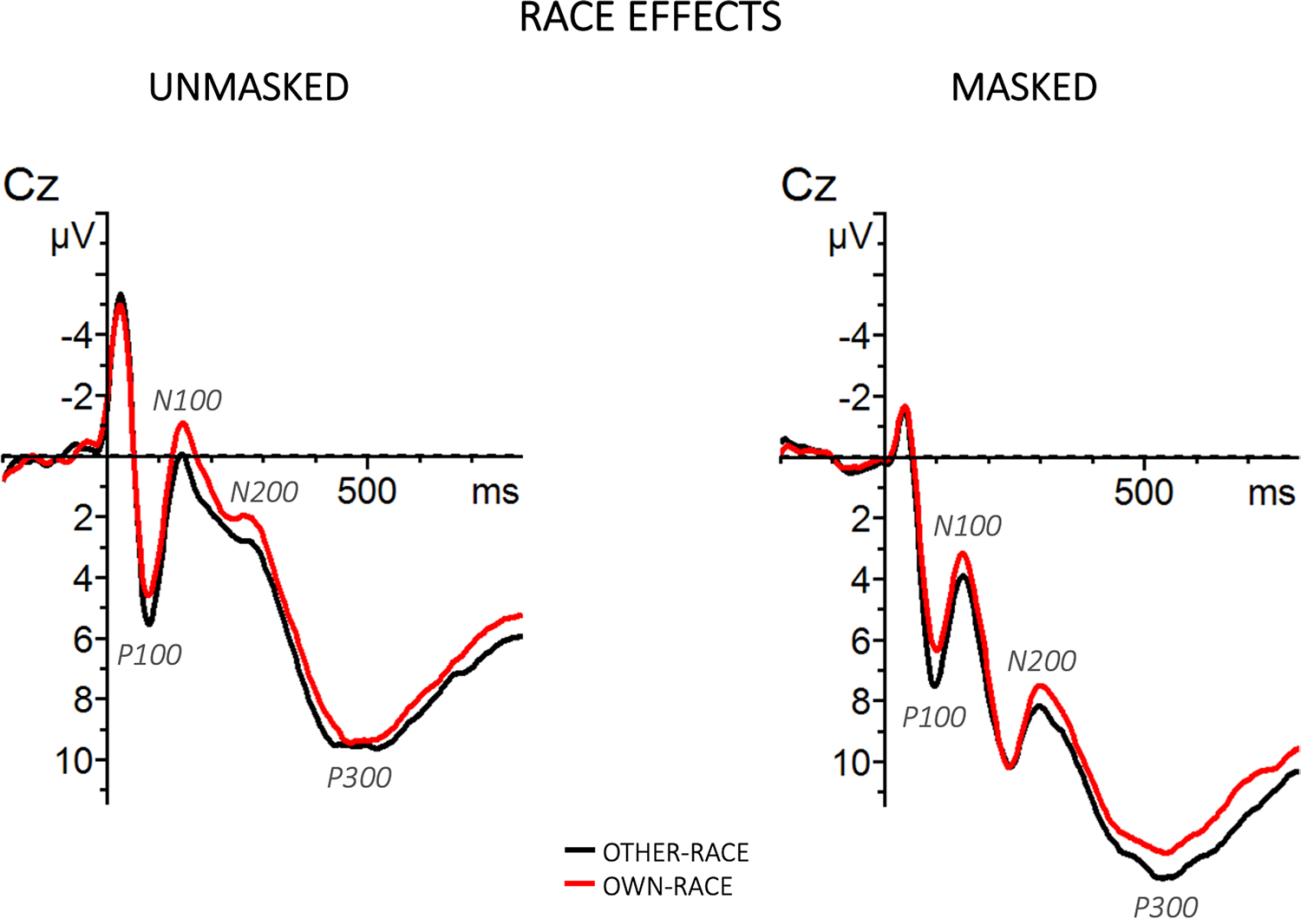
Grand-averaged ERP waveforms elicited by target faces separately for the unmasked (left panel) and masked (right panel) procedures as a function of race (other-race vs own-race).

**Figure 3:**
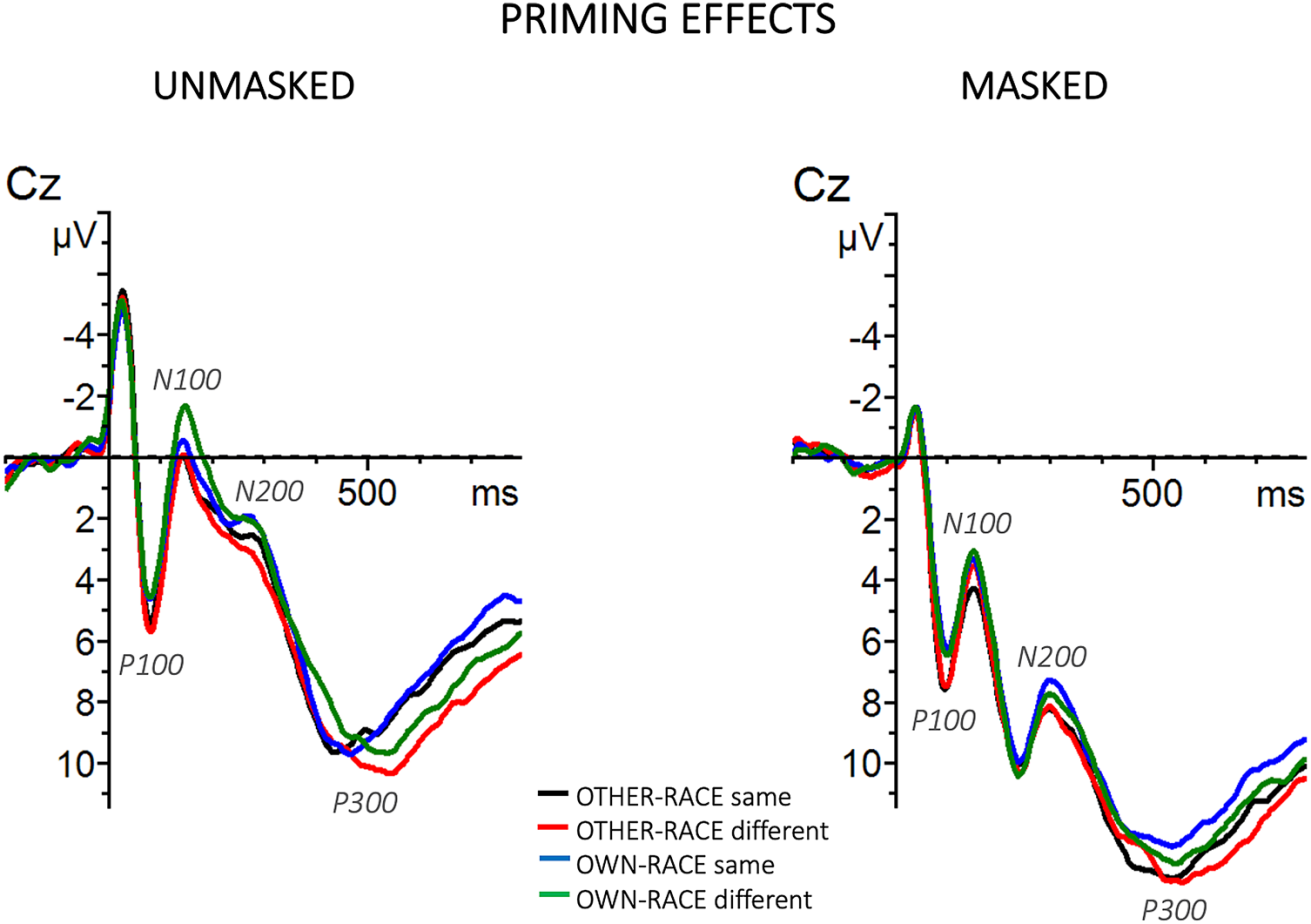
Grand-averaged ERP waveforms elicited by target faces separately for the unmasked (left panel) and masked (right panel) procedures as a function of prime-target identity (same vs different) and race (other-race vs own-race).

**Figure 4:**
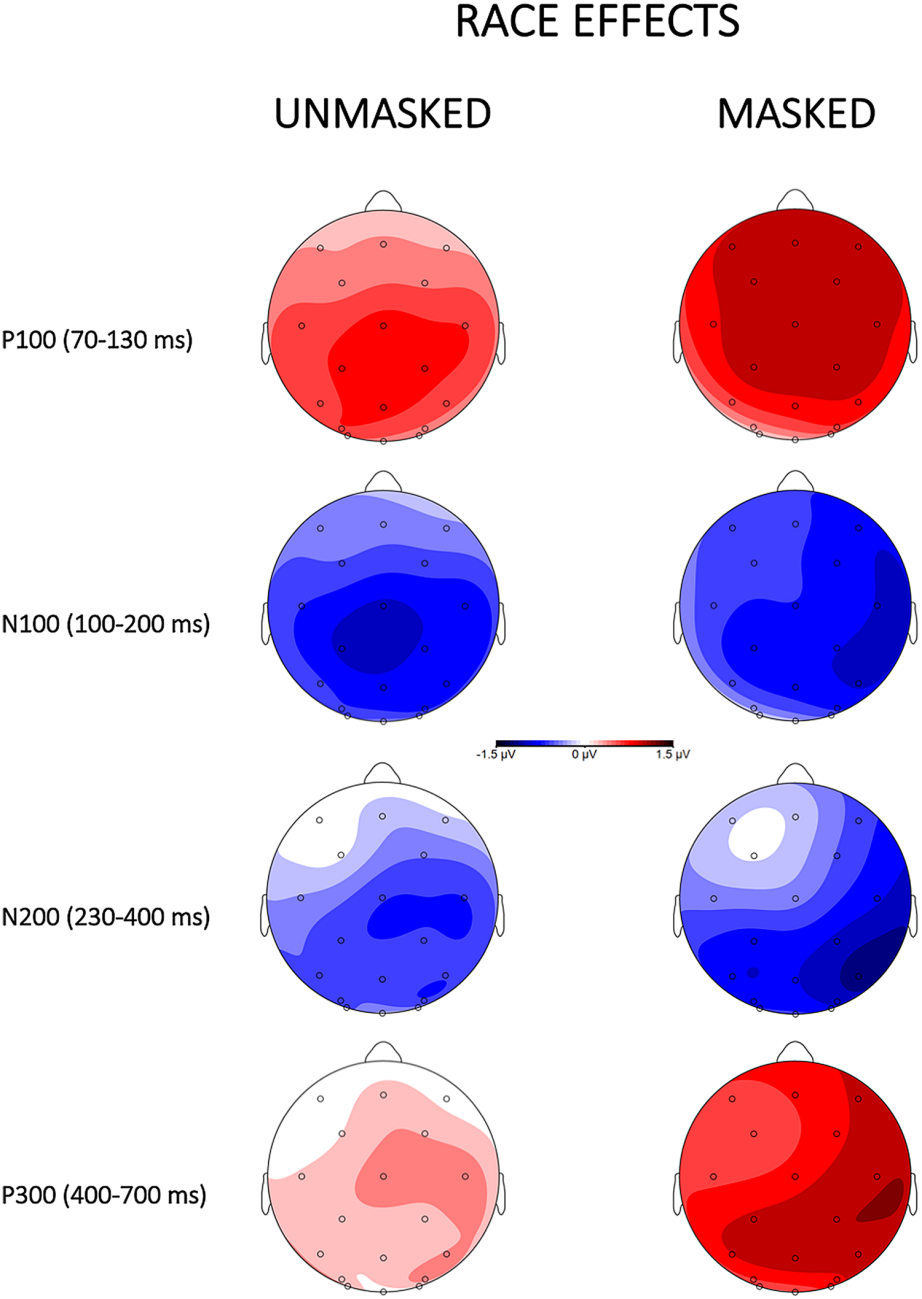
Topographical scalp distributions for target faces separately for the unmasked (left panel) and masked (right panel) procedures as a function of race (other-race vs own-race) in the four critical time windows, created by subtracting other from own-race conditions.

**Figure 5:**
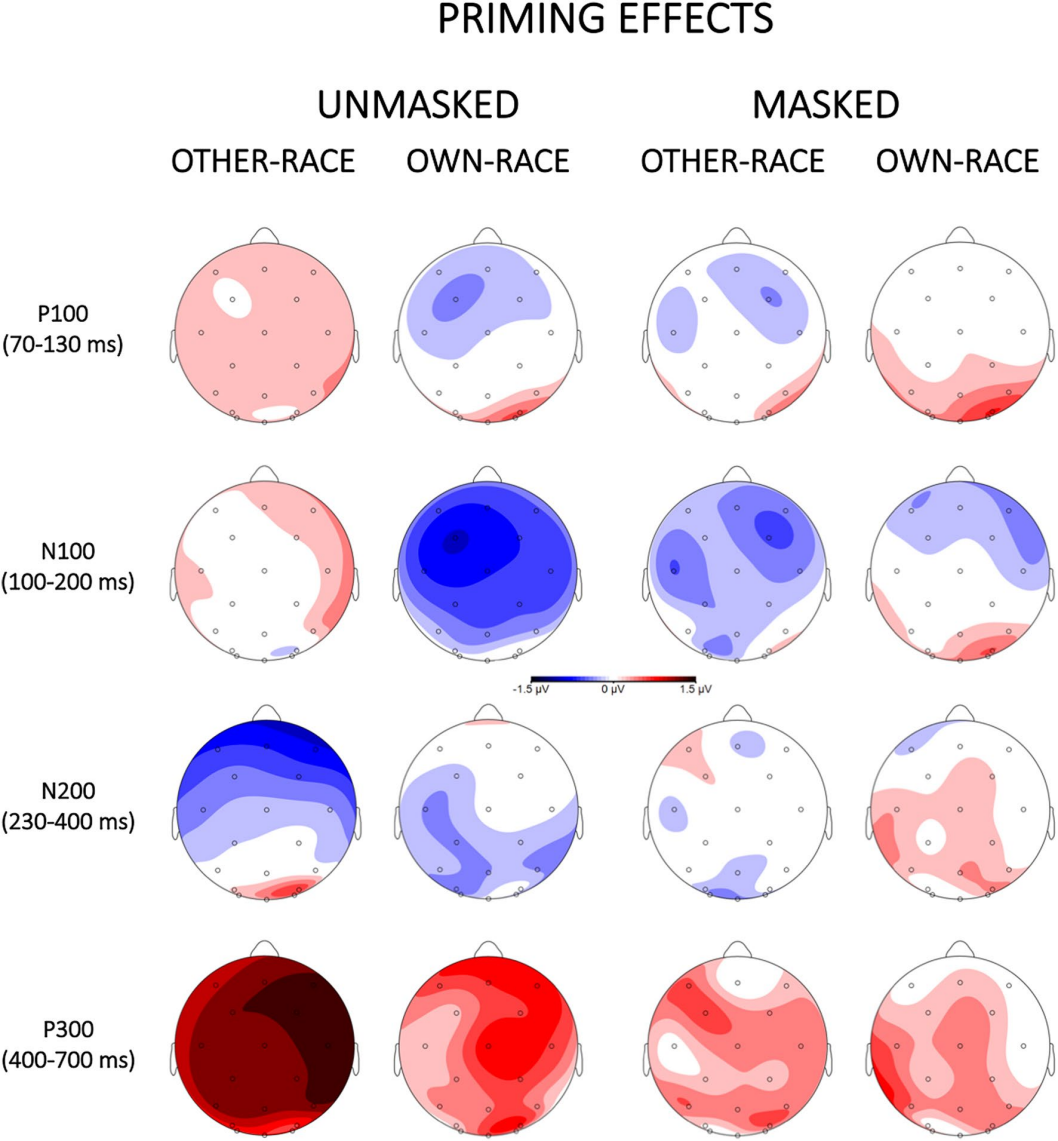
Topographical scalp distributions for target face separately for the unmasked (left panel) and masked (right panel) procedures as a function of a function of prime-target identity (same vs different) and race (other-race vs own-race) in the four critical time windows, created by subtracting same other-race and own-race conditions from different other-race and own-race ones, respectively.

Grand-averaged ERPs elicited by the different experimental conditions are represented in Figs. 2, 3, 4, and 5. Visual inspection revealed that, in all components, the magnitude of the effects was maximal at the Cz electrode, thus, for an easier visualization of our results, we show only the Cz electrode.

#### P100

The omnibus ANOVA yielded significant main effects of Masking [F(1, 34) = 7.97, p < 0.01, η_p_^2^ = 0.19], with more positive waveforms for the masked (μV = 4.57, SE = 0.54) than for the unmasked condition (μV = 3.14, SE = 0.54), of Race [F(1, 34) = 48.24, p < 0.001, η_p_^2^ = 0.59], indicating a more positive brain response for other-race (μV = 4.25, SE = 0.48) than own-race faces (μV = 3.46, SE = 0.48). No other main effects and interactions of interest to the study reached significance (ps > 0.1).

#### N100

The P100 was followed by a negative waveform identified as an N100 component. The omnibus ANOVA revealed a main effect of Masking [F(1, 34) = 24.23, p < 0.001, η_p_^2^ = 0.42], with more negative waveforms for the unmasked (μV = 1.79, SE = 0.51) than for the masked (μV = 4.26, SE = 0.51) condition, and a main effect of Race [F(1, 34) = 40.05, p < 0.001, η_p_^2^ = 0.54], with more negative waveforms for the own-race (μV = 2.65, SE = 0.45) than for the other-race (μV = 3.40, SE = 0.45) condition. The ANOVA also showed a Masking x Race x Identity interaction [F(1, 34) = 12.04, p < 0.001, η_p_^2^ = 0.26], showing a priming effect (more negative waveforms for different than same conditions) for own-race faces (different μV = 1.12, SE = 0.53; same μV = 1.73, SE = 0.53) and not for other-race faces (different μV = 2.29, SE = 0.53; same μV = 2.0, SE = 0.53) and only in the unmasked condition. No other main effects and interactions of interest to the study reached significance (ps > 0.1).

#### N200

The omnibus ANOVA showed significant main effects of Masking [F(1, 34) = 33.15, p < 0.001, η_p_^2^ = 0.49], with more negative waveforms for the unmasked (μV = 5.07, SE = 0.69) than for the masked (μV = 8.40, SE = 0.69) condition, and of Race [F(1, 34) = 16.78, p < 0.001, η_p_^2^ = 0.33], with more negative waveforms for the own-race (μV = 6.44, SE = 0.63) than for the other-race (μV = 7.03, SE = 0.63) condition. The ANOVA also showed the following significant interactions: Latitude x Masking x Race [F(1.92, 65.15) = 3.21, p < 0.05, η_p_^2^ = 0.09], Longitude x Masking x Identity [F(1.19, 40.39) = 4.59, p < 0.05, η_p_^2^ = 0.12], and Latitude x Longitude x Masking x Race x Identity [F(3.71, 126.02) = 3.72, p < 0.01, η_p_^2^ = 0.10]. To further explore these interactions, the unmasked and masked conditions were analyzed separately. The analysis of the unmasked condition showed a significant main effect of Race [F(1, 34) = 5.23, p < 0.05, η_p_^2^ = 0.13], with more negative brain response to own-race (μV = 4.94, SE = 0.76) than to other-race (μV = 5.44, SE = 0.76) faces, a significant Longitude x Race interaction [F(1.6, 54.58) = 7.31, p < 0.001, η_p_^2^ = 0.18], indicating a race effect more pronounced in the centro-parietal area (anterior: own-race μV = 1.15, SE = 0.85, other-race μV = 1.36, SE = 0.85; central: own-race μV = 4.44, SE = 0.85, other-race μV = 5.07, SE = 0.85; parietal: own-race μV = 9.24, SE = 0.85, other-race μV = 9.89, SE = 0.85), and a significant Latitude x Longitude x Race x Identity interaction [F(2.9, 98.53) = 3.29, p < 0.05, η_p_^2^ = 0.09] indicating an unmasked priming effect for the other-race condition in the anterior-left-midline area (anterior-left: different μV = 1.43, SE = 0.89, same μV = 0.74, SE = 0.89; anterior-midline: different μV = 1.29, SE = 0.89, same μV = 0.57, SE = 0.89). In the masked condition a more negative waveform for the own-race (μV = 8.07, SE = 0.59) than for the other-race (μV = 8.74, SE = 0.59) race emerged, as suggested by a significant main effect of Race [F(1, 34) = 11.10, p < 0.01, η_p_^2^ = 0.25]. Moreover, the analysis showed a significant Latitude x Race interaction [F(1.7, 57.72) = 10.03, p < 0.001, η_p_^2^ = 0.23], indicating a race effect more pronounced in the right area (left: own-race μV = 7.69, SE = 0.6, other-race μV = 8.22, SE = 0.6; midline: own-race μV = 8.72, SE = 0.6, other-race μV = 9.27, SE = 0.6; right: own-race μV = 7.8, SE = 0.6, other-race μV = 8.72, SE = 0.6). No other main effects and interactions of interest to the study reached significance (ps > 0.1).

#### P300

The omnibus ANOVA revealed a significant main effect of Masking [F(1, 34) = 28.77, p < 0.01, η_p_^2^ = 0.46], with more positive waveforms for the masked (μV = 10.51, SE = 0.73) than for the unmasked (μV = 7.48, SE = 0.73) condition, of Race [F(1, 34) = 16.63, p < 0.001, η_p_^2^ = 0.33], with more positive waveforms for other-race (μV = 9.29, SE = 0.67) than for the own-race (μV = 8.70, SE = 0.67) condition, and of Identity [F(1, 34) = 11.36, p < 0.01, η_p_^2^ = 0.25], indicating a more positive brain response for the different (μV = 9.25, SE = 0.67) than the same (μV = 8.74, SE = 0.67) condition. The ANOVA also sowed a Latitude x Race interaction [F(1.9, 63.51) = 4.04, p < 0.02, η_p_^2^ = 0.11], indicating a race effect more pronounced in the right hemisphere (left: own-race μV = 8.14, SE = 0.69, other-race μV = 8.61, SE = 0.69; midline: own-race μV = 9.26, SE = 0.69, other-race μV = 9.86, SE = 0.69; right: own-race μV = 8.72, SE = 0.69, other-race μV = 9.39, SE = 0.69), and a Masking x Race x Identity interaction [F(1, 34) = 5.10, p < 0.05, η_p_^2^ = 0.13], indicating a larger priming effect (more positive waveforms for different than same conditions) for other-race than own-race stimuli more pronounced in the unmasked (other-race: different μV = 8.24, SE = 0.75; same μV = 6.98, SE = 0.75; own-race: different μV = 7.52, SE = 0.75; same μV = 7.17, SE = 0.75) than masked (other-race: different μV = 11.01, SE = 0.75; same μV = 10.92, SE = 0.75; own-race: different μV = 10.22, SE = 0.75; same μV = 9.90, SE = 0.75) condition. No other interactions of interest to the study reached significance (ps > 0.1).

## Discussion

The purpose of our study was to investigate the neural mechanisms underlying race processing, and whether and to which extent these processes occurred outside of people’s awareness. In the present work, participants had to report the gender of the face target ignoring the face prime that was displayed masked or unmasked. In line with the literature, the behavioral findings of the present study highlighted a race effect: slower reaction times for other-race faces than for own-race faces, regardless of masking (e.g.^43^).

Moreover, race effects were seen on all ERP components analyzed (P100, N100, N200, and P300), in both the unmasked and masked conditions. The ERP findings showed rather early race effects: a larger P100 amplitude for other-race compared to own-race faces^44^, and a more negative N100 waveform for own-race than for other-race faces. These findings, are in line with the literature^23,25,26,31^ and seem to highlight that people are able, in the earliest stages of face processing, to distinguish between a face belonging to their own or other ethnic groups. These effects on the P100 and N100 agree with previous ERP evidence suggesting that information related to race is available very early before perceptual processes are concluded. We also found an N200 race modulation, with more negative amplitudes for own-race faces than other-race faces. These N100 and N200 enhanced negativities for own-race faces can be interpreted, as suggested by previous works, as a reflection of an automatic shifting of attention to ingroup (own-race) faces for a more in deep analysis following early greater attention to outgroup (other-race) members in the P100^25,26,32,33^.

In our study, the race also modulated the amplitude of a later component such as the P300. The analyzes on the P300 showed a larger positivity for other-race than own-race faces, confirming the data present in the literature on the involvement of this component for faces belonging to another race or belonging to a different social category^26,45,46^. It is worth noting that in our research an implicit task has been used (to attend to gender and not explicitly to race), this means that all our race effects reported above occurred regardless of whether the participants were explicitly classifying the faces in terms of race, thus, all our results index implicit and automatic processing of race.

While race effects were seen in all ERP components analyzed (P100, N100, N200, and P300) and regardless of masking, priming effects as a function of race, were seen on the N100, N200, and P300 components, and only in the latter in the masked condition. It is worth noting that, the first waveforms to be modulated by the interaction between prime and target as a function of race, was the N100, being larger when the stimuli were different than same for own-race faces and only in the unmasked condition. This N100 unmasked priming effect, suggests an automatic brain mechanism underlying an early recognition of faces and seems in accordance with previous N100 findings that have been suggesting that information relative to race is available early in face processing, even before perceptual processing are complete (^26^; but see^25^). Given that, this early component has been related to the automatic allocation of attention in response to attention-grabbing stimuli^47^, our early N100 priming effect for own-race stimuli can be interpreted as a reflection of initially greater attention to stimuli that did match (e.g., for identity) the preceding context of more familiar (own-race) stimuli. Or it could be interpreted as individuation-related features being more effectively processed for own-race than other-race faces in early processing stages, since the N100 was sensitive to incongruency between a target and its prime in own-race but not in other-race faces.

Moreover, we also found an unmasked priming effect on the N200 component, with larger negativity for same than different trials for other-race and not for own-race faces. This priming effect (larger negativity for same stimuli) is in line with many repetition and semantic priming studies where has been found a larger N200 amplitude for repeated and semantically congruent stimuli (e.g.^48,49^). The fact that this priming effect is present only for other-race faces can be interpreted as reflecting a more in deep processing of unfamiliar (outgroup) stimuli. It could reflect a facilitated processing of other-race target faces by the pre-activation of their memory representation at this processing stage, since this representation could be difficult to access or less precise as compared to the one for own-race faces. Thus, it could index a more difficult processing of other-race faces.

The last component affected by priming was the P300. Interestingly, the P300 was the only component where a priming effect as a function of race was found not only in the unmasked condition but also in the masked condition and both for own-race and other-race faces. This enhanced P300 observed for different relative to same targets in both masked and unmasked conditions might indicate an automatic and implicit brain mechanism underlying the recognition and identification of faces. Interestingly to note, the priming effect was larger for other-race faces and more pronounced in the unmasked condition, showing a facilitated processing of stimuli that matched a pre-existing memory representation (own-race) and a more in deep processing of unfamiliar (outgroup) stimuli (started in the N200 time window). In other words, greater difficulty to process faces belonging to other-race. It is worthy of note that, although the N100 and the N200 were not sensitive to the relation between the prime and the target face as a function of race in the masked condition, the P300 component did change as a function of the preceding prime face in both unmasked and masked conditions. The P300 is thought to reflect the updating of information in working memory^39,50^. Accordingly, our findings have thus been taken as evidence that working memory processes were affected by social category information in a context in which race categorization was occurring implicitly (gender task) and the stimuli were presented below threshold. Overall, the P300 effect in the masked condition showed that the race of a face could be processed automatically and unconsciously, i.e., outside of conscious awareness.

Our results indicate that at the earlier stage (P100) of processing, effects were mainly accountable to race properties of the stimulus, regardless of the more complex relation between the prime and the target stimulus. While later stages (N100, N200, P300) are sensitive to these latter effects.

Taken together our findings further confirm that race information is present remarkably early in face processing. More importantly, we provided for the first time evidence that these effects occur even without conscious inferences. Indeed, our P300 masked priming modulation on face targets preceded by face primes in both other-race and own-race conditions can only be attributable to participants having not consciously processed the face primes, confirming and extending the hypothesis that the activation of race information is automatic and occurs very early in time. Conclusively, our results cannot fit with a strand of the sociocognitive theory arguing for a strategically or intentionally deeper processing of own-race faces (e.g.^5,17^) because our race-priming effects occurred automatically and even without conscious inferences. On the contrary, our results fit with the EBH account of the ORE, because they seem to reflect a different accessibility or matching difficulty between incoming visual information and memory representations between other-race and own-race faces in Caucasian observers. Our results could also be interpreted from other sociocognitive points of view. For instance, it is possible that participants automatically categorized own-race faces as ingroup and other-race faces as outgroup, and that different priming effects for different races were a result of the automatic processing of identity-specifying features in own-race faces and race-specifying features in other-race faces^51,52^. This could explain situations in which the priming effect is present for own-race but not for other-race faces, since the effect is based on identity repetition. This account is also in line with early ERP effects that we observed for face race (e.g., on the P100 and N100), which could be interpreted as reflecting rapid social categorization. However, greater priming effects for other-race faces (see e.g., N200 priming effects) are difficult to interpret within this framework, because since identity here is not task relevant, there would be no motivation for the observer to put more effort and go deeper (to the individual level) in the processing of other-race faces. Finally, these effects could also be explained as a matter of differences in attention capture: own-race faces could automatically capture more attention than other-race faces, but own-race faces would eventually be attended to more than other-race faces. This could be reflected by the pattern of the P100, N100, and N200 sensitivity to own- and other-race faces. The effects of priming as a function of race could be interpreted as more attention being allocated to changes of identity between target and prime face in own-race than in other-race faces. However, the account could not explain the opposite effects.

To our knowledge, none of the studies examined the brain correlates and the temporal characteristics of implicit race processing by comparing face stimuli above and below threshold. Our study adds further evidence that race information influences face processing, but unlike works already present in the literature, our data clearly highlights how this influence is immediate and automatic. Our research suggests that race information grabs attention automatically and quickly, at early processing stages. Overall, the data from this study seems to support the hypothesis for which race processing is a rather early process, also found in conditions of unconsciousness. To note, the present research was limited to Caucasian participants and the ORE was observed in a specific context (Asian faces vs Caucasian faces). Future studies should further investigate whether our research can be generalized to faces of other races and other ethnic samples.

## Method

### Ethics statement

This study was carried out following the recommendations of the “Italian Association of Psychology” (AIP) Ethical Guidelines (Codice Etico: www.aipass.org/node/11560), was reviewed and received formal approval by the local Ethical Committee of the Host institution of the second author (School of Psychology of the University of Padua-Italy). Participants were informed of their rights and gave written informed consent for participation in the study, according to the Declaration of Helsinki. All study procedures met the ethical guidelines for the protection of human participants, including adherence to the legal requirements of the Country.

### Participants

Thirty-five students at the University of Modena and Reggio Emilia with Caucasian ethnic backgrounds (18 women; age range: 19–27 yrs, M = 22 yrs) participated in the experiment. All participants were right-handed (L.Q. =  + 88, Decile R.7) as assessed with an Italian version of the Edinburgh Handedness Inventory^53^. Participants had no history of neurological or mental disorders and had normal or corrected-to-normal visual acuity. The criteria for considering participants as Caucasians are: born in western countries, white-skinned and with Caucasian parents, and no Asian (the ethnic group not belonging to their own) relatives or close friends.

### Stimuli

88 pictures of faces were used, 44 Caucasian and 44 Asian faces, 50% of each female; each picture was displayed four times, for a total of 352 stimuli. Face pictures were selected from the FERET database (FERET Facial Image Database Release 2^54^). Asian pictures rather than African-American have been used given that Asian and Caucasian pictures have similar luminance and contrast. However, they were imported into Adobe Photoshop, luminance was controlled within each racial group (Asian = 121 mean luminance, Caucasian = 123 mean luminance; p > 0.1).

The background and all of the details of the face (as ears, hair, and neck) were hidden (covered) using a black oval passe-partout. Two separate surveys to control race and gender reliability of the experimental face stimuli were each presented to 60 participants. In the race survey was asked to rate the extent to which each face was associated with a Caucasian or Asian face, while in the gender survey to rate the extent to which each face was associated with a male or female face. For both surveys, a seven-point Likert scale has been used (half of the participants saw: 1 -Asian/Male and 7 -Caucasian/Female, the other half saw a reversed scale). The final rating assigned to each face was calculated by combining the ratings obtained with both directions of the rating scale. All experimental face stimuli emerged to be prototypical of both race and gender. The overall average for Asian faces was 1.09 (SD = 0.12, range 1–2), for Caucasian faces was 6.95 (SD = 0.1, range 6–7), for Male faces was 1.01 (SD = 0.05, range 1–2), and for Female faces was 6.97 (SD = 0.12, range 6–7). Two types of prime-target pair faces were used: Same face pairs (prime-target were identical faces), and Different face pairs (prime-target were different faces). Prime-target pairs were half Asian (other-race) and half Caucasian (own-race) faces. The face stimuli on each pair belonged to the same gender and race. Four are the resulting conditions: 1. Other-race Same; 2. Other-race Different; 3. Own-race Same; 4. Own-race Different. The prime was 25% smaller than the target to avoid any perceptual overlapping.

In the present work, we used a masked and unmasked priming paradigm highly similar to that employed by Pesciarelli, et al.^55^, (see “Method” section), with the difference that in the present study we presented human faces as prime-target pairs instead of words. In each trial of the masked condition, the prime face was preceded and followed by masking stimuli. In each trial of the unmasked condition, the masking stimuli were replaced by a black screen of the same duration of the mask. Participants performed eight blocks of 88 trials each, resulting in a total of 704 trials (44 trials per condition). To avoid revealing the presence of the prime stimulus the first blocks were masked and the last four unmasked. Within each block, the four conditions (Other-race Same; Other-race Different; Own-race Same; Own-race Different) appeared in randomized order and with the same probability. The critical stimuli on which ERP data were compared were the target faces of each trial. Prime-target pairs were randomized before presentation. Before the experiment, participants took part in a short training session with 16 prime-target pairs (8 masked and 8 unmasked, half Caucasian and half Asian) formed by stimuli different from the experimental ones. The masking stimulus consisted of a scrambled picture of a face with the same luminance and contrast of the prime and target and the same dimensions and visual angle of the target.

### Design and procedure

**Figure 6:**
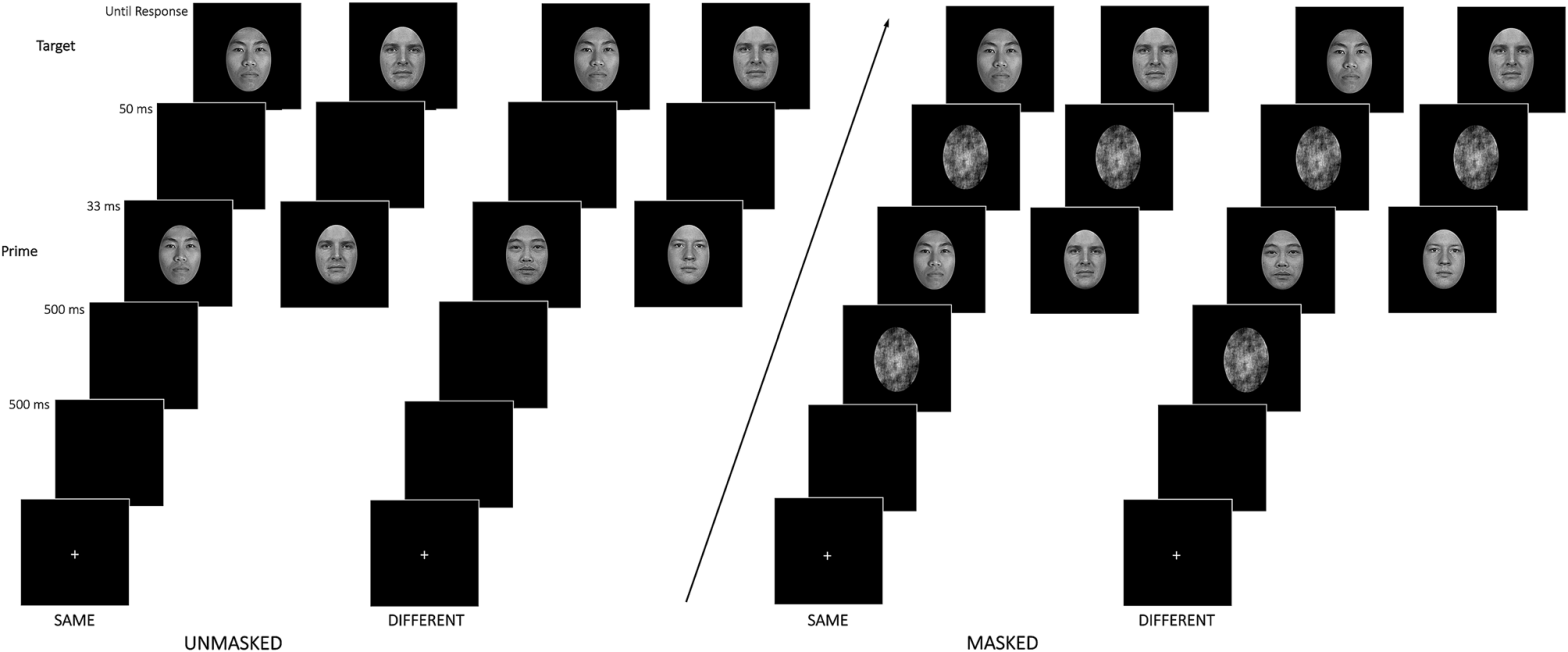
Schematic depiction of the unmasked and masked procedures used in the present experiment.

Participants were seated comfortably in a darkened sound-attenuated room. An example of the stimulus presentation procedure is illustrated in Fig. 6.

As in Pesciarelli et al.^55^, (see “Method” section), all stimuli were presented in the center of a 17″ CRT monitor synchronous with the screen refresh [Philips 107B; refresh rate = 60 Hz (16.67 ms)] that was positioned at eye level approximately 70 cm in front of the participant, such that each target and mask stimulus subtended 11.3° of visual angle. The prime stimulus was 25% smaller (visual angle 8.5°) than the target stimulus, to avoid any perceptual overlapping. Stimuli were displayed against a black background. E-Prime software (Version 2; Psychology Software Tools, Pittsburgh, PA) was used for stimulus presentation and behavioral response collection. The test computer was an ACPI multiprocessor PC with a D CPU 2.80 GHz Intel Pentium processor, Radeon X550 video card. Priority settings were optimized to ensure accurate display durations. Each trial began with a fixation cross (+) presented in the middle of the screen and stayed there until participants pressed a button to start the trial. Then a black screen was displayed for 500 ms and replaced by a 500 ms forward mask consisting of a scrambled picture of a face. The forward mask was replaced by the prime stimulus for 33 ms at the same location on the screen. The use of a 33 ms prime stimulus duration was expected to make masking effective and to prevent participants from consciously perceiving the primes. The prime was then immediately followed by a 50 ms backward mask consisting of a scrambled face. Then the target face appeared and remained on the screen until a response was made. Each response was followed by a 1000 ms blank screen. The use of prolonged forward and backward mask durations was expected to make masking even more effective preventing participants from consciously perceiving the primes and avoiding selection bias.

In the unmasked condition, the masking stimuli were replaced by a black screen of the same duration as the mask. This manipulation made the prime stimulus fully visible, above threshold.

The task of the participants was to decide, as quickly and accurately as possible, whether the target was a female or a male face (gender task). Participants responded by pressing one of two buttons, which were counterbalanced (left and right) across participants. This implicit task (to attend to gender and not explicitly to race) has been used to assess responses as a function of race to index the degree to which race is automatically processed.

An objective measure of prime visibility was obtained after the experiment for the masked condition. Participants were informed of the presence of the prime behind the masks and had to perform a gender task on masked prime faces that could be either a female or a male. They received a practice session to ensure that they understood the prime visibility task. Participants were also requested to make the best guess when they felt not confident about the correct response. Data of no participants had to be excluded from the analysis because the identification rate did not exceed the confidence interval of chance performance (accuracy greater than 70%). The gender task on the masked primes confirmed that our masking method rendered the primes largely invisible, as the average accuracy was close to chance [mean percentage correct = 55% (SD = 0.10, range 35–70%). Accuracy was distributed around the chance level of 50%, which is expected by mere guessing. This objective prime visibility measure overcomes the limitations of subjective self-report measure in which participants report not having seen the stimuli but may have experienced it consciously^55^.

### EEG recording and analysis

As in Pesciarelli et al.^55^, (see “Method” section), EEG was amplified and recorded with the BioSemi Active-Two System from 30 active electrodes placed on the scalp according to the International 10–20 System^56^. Besides, four electrodes were placed around the eyes for eye-movement monitoring (two at the external ocular canthi and two below the eyes) and two electrodes were placed over the left and right mastoids. Two additional electrodes were placed close to Cz, the Common Mode Sense [CMS] active electrode and the Driven Right Leg [DRL] passive electrode, which were used to form the feedback loop that drives the average potential of the participant as close as possible to the AD-box reference potential. EEG and EOG signals were amplified and digitized continuously with a sampling rate of 512 Hz. Brain Vision Analyzer (Brain Products, Gilching, Germany) was used to perform off-line signal processing analyses. EEG signal was bandpass filtered between 0.01 and 80 Hz and referenced off-line to the average activity of the two mastoids. Artifact activity was rejected using a semiautomated procedure, with artifacts identified by the following criteria: Gradient, with 25 μV maximal allowed voltage step; Max–Min with 200 ms maximal allowed absolute difference; Low activity, with 0.5 μV/50 ms lowest allowed activity. Data with excessive blinks were adaptively corrected using ICA. 1200-ms epochs containing the ERP elicited by the target face were extracted, starting with 200 ms before the onset of the face. ﻿A 200 ms pre-stimulus baseline was used in all analyses. Segments including artifacts due to activity exceeding ± 100 μV in amplitude were also rejected. The lost data (due to artifacts) of the 35 participants were equal to 4.7%. Overall, averaged ERPs included: in the masked manipulation, an average of 69.8 trails for the Other-race same, 70.1 for the Other-race different, 73.3 for the Own-race same, 73.4 for the Own-race different conditions and in the unmasked manipulation, an average of 71.5 trails per the Other-race same; 71.2 Other-race different; 71.7 Own-race same; 72.9 Own-race different conditions. The averaged ERPs were low-pass filtered at 30 Hz. Based on visual inspection of grand average ERP waveforms and in line with our previous studies and previous literature^15,44,49,57,58^, the following components were identified for target onset at frontal (F3, Fz, F4), central (C3, Cz, C4), and parietal (P3, Pz, P4) scalp sites: P100 from 70 to 130 ms after target onset; N100 from 100 to 200 ms after target onset; N200 from 230 to 400 ms after target onset; P300 from 400 to 700 ms after target onset. For each ERP component amplitude was measured as mean activity within the respective time window.

### Statistical analysis

Behavioral and ERP analyses were carried out only on trials with correct responses. ﻿Individual reaction times (RTs) exceeding ± 2 SD were eliminated (4.4%). The mean response times (RTs) of correct responses per condition were submitted to analyses of variance (ANOVAs) with Masking (unmasked, masked), Race (other-race, own-race), Identity (Same, Different), as within-subject factors. ERP effects time-locked to the onset of the target were evaluated considering 6 clusters of electrodes representing the mean amplitude of three electrodes in close position: Anterior (F3, Fz, F4), Central (C3, Cz, C4), Posterior (P3, Pz, P4), Left (F3, C3, P3), Midline (Fz, Cz, Pz), Right (F4, C4, P4). ANOVAs were conducted on mean ERP amplitudes with Masking (unmasked, masked), Race (other-race, own-race), Identity (same, different), Longitude (anterior, central, posterior), and Latitude (left, midline, right) as within-subject factors. When appropriate, degrees of freedom were adjusted according to the method of Greenhouse–Geisser; only corrected significance levels are reported. The level of significance testing was p = 0.05. The main effects of Masking, Identity, and Electrode position are not central to the questions under study. Therefore, they are reported and not discussed since we discuss only the main effects and interactions of interest to the study. Significant ERP effects on Longitude and Latitude factors are reported in the “Appendix”.

## Supplementary Information

Supplementary Information.

## Data Availability

Data will be made available upon request. Requests may be emailed to F.P. at francesca.pesciarelli@unimore.it.
