# Supplementary material for: Electrophysiological correlates of unconscious processes of race
Source: Sci Rep. 2021 Jun 2;11:11646. doi: 10.1038/s41598-021-91133-2 (PMC8172900; doi:10.1038/s41598-021-91133-2)
Supplement: Supplementary file 1 — Supplementary Information. [file 41598_2021_91133_MOESM1_ESM.docx]

| **ERP components Factorial level *F* value *p* value *ηp*^2^** |
| --- |
| P100 latitude 22.38 < .001 .4  longitude 4.79 < .05 .12  latitude x longitude 8.02 < .001 .19  N200 latitude 6.74 < .01 .16  longitude 41.79 < .001 .55  latitude x longitude 11.64 < .001 .26  P300 latitude 11.45 < .001 .25  longitude 80.69 < .001 .7  latitude x longitude 7.87 < .001 .19 |

Appendix. Significant ERP effects on Longitude and Latitude factors.
